# Weichang’an Formula Inhibits Tumor Growth in Combination with Bevacizumab in a Murine Model of Colon Cancer—Making up for the Deficiency of Bevacizumab by inhibiting VEGFR-1

**DOI:** 10.3389/fphar.2020.512598

**Published:** 2020-11-30

**Authors:** Chuan-Fang Pan, Xi Zhang, Jing-Wen Wang, Tao Yang, Linda L. D. Zhong, Ke-Ping Shen

**Affiliations:** ^1^ Shanghai University of Traditional Chinese Medicine, Shanghai, China; ^2^ School of Nursing, Shengli College, China University of Petroleum, Dongying, China; ^3^ Hong Kong Baptist University, Kowloon, Hong Kong

**Keywords:** traditional Chinese medicine, cancer, angiogenesis, Weichang’An formula, leptin/STAT3 signal pathway, immunity

## Abstract

**Aim:** Angiogenesis plays an important role in the initiation, development, and metastasis of malignant tumors. Antiangiogenic drugs combined with immune therapy are considered to have a synergistic effect on anti-tumor strategy. Weichang’an formula (WCAF) is a prescription of traditional Chinese medicine (TCM) based on pharmaceutical screening and clinical experience. The aim of this study is to examine the effect of WCAF and its combined action with Bevacizumab (BEV) in colorectal cancer, and to identify the possible mechanism of action.

**Methods:** A human colon cancer cell (HCT 116) subcutaneous xenograft model was established in BALB/c-nu/nu mice. Tumor-bearing mice were randomized into each of four groups: control, WCAF treated, BEV treated, and WCAF plus BEV treated. Apoptosis was detected by TUNEL assay. Western blot was used to assess the protein levels of Leptin-R, STAT3, p-STAT3, BCL-2, and VEGFR-1. Immunohistochemistry was used to detect the micro-vessel density (MVD) and AKT1. Leptin and Vascular endothelial growth factor A (VEGF-A) mRNA expression were detected by Real-time PCR (RT-PCR). A network pharmacology study and validation assay were carried out to find the underlying molecular targets of WCAF related to immune regulation.

**Results:** Compared with the control group, WCAF reduced tumor weight and volume, as well as promoted tumor cell apoptosis. WCAF treatment decreased the mRNA expression of Leptin and *VEGF-A,* while the protein levels of CD31, LEP-R, VEGFR-1, STAT3, and p-STAT3 were decreased in tumor tissues. In addition, VEGFR-1 protein expression was decreased in the WCAF group and the WCAF plus BEV group but not in the BEV group. The combination of WCAF and BEV demonstrated a partial additive anti-tumor effect *in vivo.* The pharmacological network also found there are 26 WCAF target proteins related to cancer immune and 12 cancer immune related pathways. The AKT1 protein expression in the WCAF and WCAF + BEV groups were significantly lower than the that in the control group (*p <* 0.01).

**Conclusion:** WCAF can inhibit tumor growth and promote apoptosis and inhibit tumor angiogenesis in subcutaneous xenografts of human colon cancer HCT-116 in nude mice. WCAF also makes up for the deficiency of BEV by inhibiting VEGFR-1. The VEGFR-1 expression between the combination group and BEV alone achieved statistically significant difference (*p* < 0.01). Combined with BEV, WCAF showed a partial additive anti-tumor effect. The mechanism may be related to Leptin/STAT3 signal transduction, VEGF-A, VEGFR-1 and WCAF target proteins related to cancer immune such as leptin and AKT1.

## Introduction

Colorectal cancer is one of the most common malignant tumors of the digestive system. It is the third most common cancer globally in men and the second most common cancer in women. Its mortality ranks fourth globally in men and third in women. Angiogenesis plays an important role in the initiation, development, and metastasis of malignant tumors (Voest and D'Amore, 2001). Inhibition of angiogenesis has been shown to be beneficial in colon cancer (Gerger et al., 2011). Vascular endothelial growth factor A (VEGF-A) is one of the major regulatory factors of tumor angiogenesis. Circulating VEGF-A binds to VEGF-receptors 1 and 2 (VEGFR-1 and VEGFR-2), stimulating the recruitment and proliferation of endothelial cells. Drugs that can inhibit VEGF production or block signaling of its receptor have shown a significant inhibitory effect on tumor growth, (Kim et al., 1993; Benjamin et al., 1999; Malonne et al., 1999). Bevacizumab (BEV), a recombinant human monoclonal antibody that binds and neutralizes circulating VEGF, has shown positive results when used in conjunction with other therapies in advanced colorectal cancer patients (Wolpin and Mayer, 2008). Combined with the first-line oxaliplatin chemotherapy, BEV significantly prolonged median progression-free survival (PFS) from 8.0 to 9.4 months in metastatic colorectal cancer. The median overall survival (OS) was also 1.4 months longer in the BEV group than in the placebo group (Saltz et al., 2008). In China, it is quite common that many colorectal cancer patients applied traditional Chinese medicine (TCM) when they are prescribed by anti-angiogenic therapy, but there are few studies on the combined application of anti-angiogenesis and TCM. According to TCM theory, Spleen Deficiency and heat-toxicity always exist in patients with digestive tract tumors (Lu and Shen, 2015). Weichang’an formula (WCAF) is a Chinese herbal formula prescribed by practitioners of TCM based on pharmaceutical screening and clinical experience. WCAF is mainly composed of some heat-clearing and toxicity-removing herbs and a classical formula (*SiJunZiTang*), which can nourish the Spleen. Previous clinical studies have shown that WCAF can reduce the recurrence and metastatic rate of colorectal and gastric cancer (Gu et al., 2006; Shen and Pan 2007). Studies have also shown that WCAF can inhibit the growth and liver metastasis of human colorectal cancer in nude mice, suppress tumor angiogenesis, and downregulate phosphorylation of signal transducer and activator of transcription 3 (STAT3) in gastric cancer cells (Zhao et al., 2007; Pan et al., 2009; Zhou and Shen, 2009; Tao et al., 2013).

In our previous study, we used subcutaneous models to investigate the effects of WCAF on gastric cancer, and we demonstrated that WCAF could inhibit colon cancer growth and liver metastasis in nude mice (Shen et al., 2012). Our *in vitro* study showed WCAF could inhibit leptin-stimulated proliferation and VEGF secretion of colon cancer HT-29 cells (Kong et al., 2019). In this study, we addressed the effects of WCAF on the growth, apoptosis, and angiogenesis of colorectal cancer by establishing a xenograft model of human colorectal cancer HCT-116 in nude mice. A network pharmacology study and validation assay were carried out to find the underlying molecular targets of WCAF related to immune regulation. We illustrated the mechanism of WCAF and discovered that its anti-tumor activity in colorectal cancer is mediated by Leptin/STAT3/VEGF signaling and its interaction with the anti-angiogenic agent BEV.

## Materials and Methods

### Materials

Fetal bovine serum, Dulbecco’s modified Eagle’s medium, penicillin, and streptomycin were purchased from Gibco (Shanghai, China). The following antibodies were used: STAT3 (79D7) Rabbit mAb (4904s), Phospho-STAT3 (Tyr705) antibody (Cell Signaling Technology, Shanghai, China, 9132s), anti-VEGF Receptor 1 antibody (ab32152), mouse VEGF-A Platinum ELISA Kit (BMS619/2), mouse Leptin ELISA Kit (ab100718), anti-Leptin Receptor antibody (Abcam, ab177469), anti-BCL-2 antibody (ab182858), and anti-CD31 antibody (Abcam, Shanghai, China, ab124432), anti-AKT1 antibody(Abcam, b59380). anti-FGF2 antibody(sc-74412). Methanol and acetonitrile (HPLC grade) were supplied by Fisher Scientific Co. (Santa Clara, United States). HPLC grade water (418 mΩ) was purified by a Milli-Q water purification system (Millipore, Milford, MA, United States). BEV was purchased from the Fudan University Affiliated Tumor Hospital. Tumor-bearing nude mice were treated with a dose of 10 mg/kg/m^2^. The concentration of BEV was adjusted to 1 mg/ml using 90% physiological saline.

### Preparation of Weichang’An Formula

**FIGURE 1:**
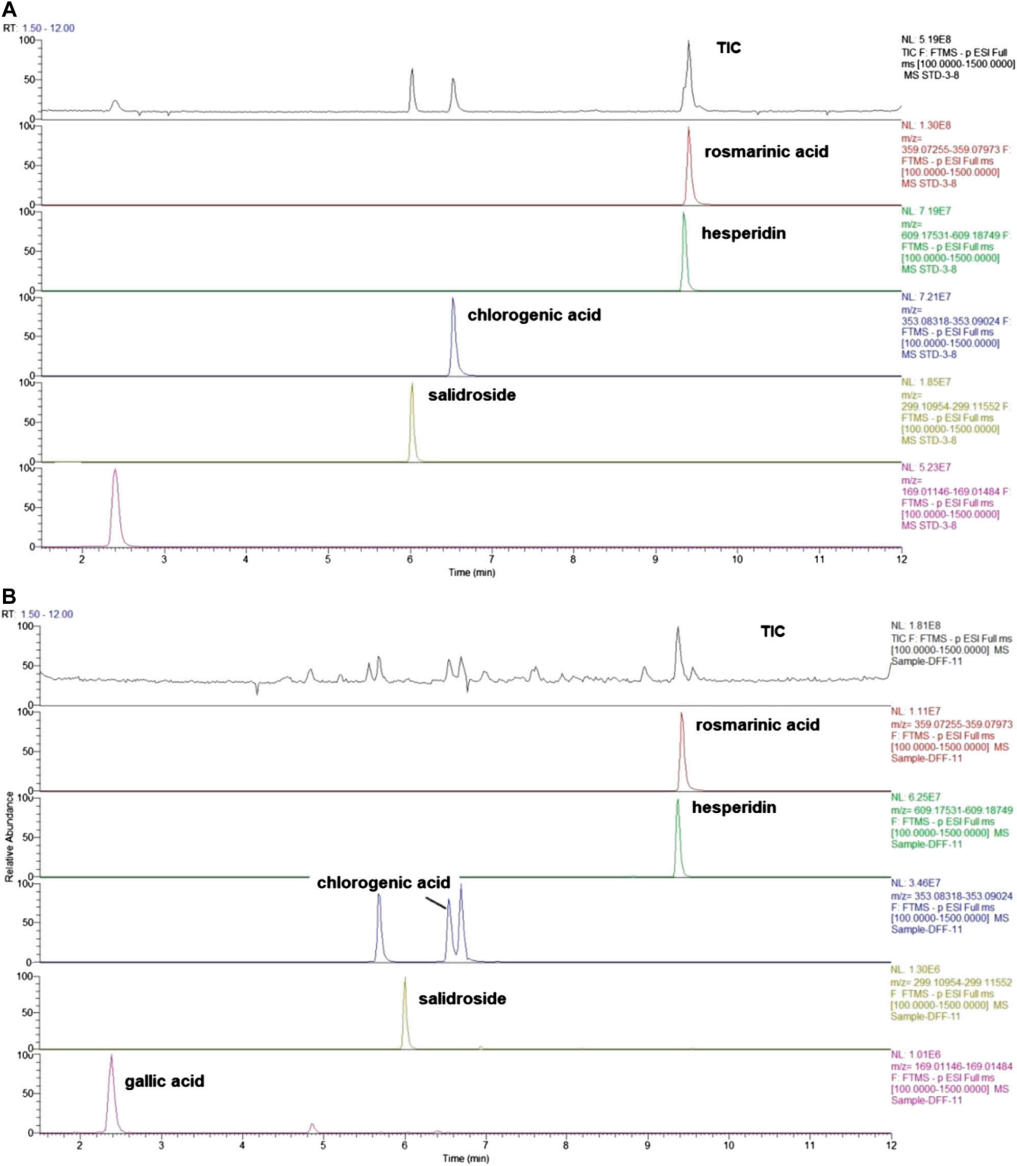
Total-ion chromatograms of standard and sample by UHPLC- Q/Exactive. **(A)** The mix standard solution. **(B)** The chromatographic profile of WCAF extracts [Stationary phase: Waters Acquity HSS T3 (100 × 2.1 mm, 1.8 μm); mobile phase: aqueous 0.1% formic acid (I) and acetonitrile (II) (0–2 min, 2%II; 2–12 min, 2–-45%II)].

WCAF is composed of Taizishen (*Pseudostellaria heterophylla (Miq.) Pax*)*,* Baishu (*Atractylodes macrocephala Koidz*), Fuling (*Wolfiporia cocos*), Daxueteng (*Sargentodoxa cuneata (Oliv.) Rehder and E.H.Wilson*), Tengligen (Actinidia arguta (Siebold and Zucc.) Planch. ex Miq. [Actinidiaceae]), Baqia (*Smilax china L*), Chenpi (*Citrus × aurantium L*), and Xiakucao (*Prunella vulgaris L*). These herbs were purchased from the Longhua Hospital Affiliated Shanghai University of TCM (Shanghai, China). All of them were identified by Dr. Tao Yang, according to the Pharmacopoeia of the People’s Republic of China (2015). Their voucher specimens were deposited at Longhua Hospital (Shanghai, China). The following components were combined as follows: Taizishen (*Pseudostellariae Radix*) 24 g, Baishu (*Atractylodis macrocephala rhizome*) 24 g, Fuling (*Poria*) 60 g, Daxueteng (*Sargentodoxae caulis*) 60 g, Baqia (*Smilax china rhizoma*) 60 g, Xiakucao (*Prunella spica*) 18 g, and Chenpi (*Citri reticulatae pericarpium*) and other crude herbs 155 g. Ten volumes of water were then added to the herb mix and soaked for 60 min. This mixture was decocted twice, once for 3 h, and then again for 2 h. The decoctions were then combined and concentrated under reduced pressure at 60 ± 2°C (1 g extract contained 6.79 g of herbal mixtures). Quality control analysis of WCAF was carried out using ultra-high performance liquid chromatography-mass spectrometry (UHPLC-Q/Exactive). The analyses were carried out with a UHPLC-Q/Exactive system (Thermo Fisher, San Jose, CA, United States) coupled with an autosampler, a quaternary gradient pump, and a quadrupole/electrostatic field orbitrap high-resolution mass spectrometry detector. The components were eluted using a gradient system consisting of aqueous 0.1% formic acid (I) and acetonitrile (II) (0–2 min, 2% II; 2–12 min, 2–45% II). Other components, including rosmarinic acid, hesperidin, chlorogenic acid, salidroside, and gallic acid, were also detected using UHPLC-Q/Exactive mass spectrometry and were present in the extracts in the amounts of 0.6824, 1.9084, 7.3586, 0.1884, and 0.1000 µg/g, respectively (Figure 1). The chromatographic proﬁle of the extracts and the compounds of the chromatographic profile of WCAF extracts were shown in Supplementary Data Sheet 1.

### Animal Models

#### Cell Culture

The human colon adenocarcinoma cell line HCT-116 was purchased from the Chinese Academy of Sciences cell bank and cultured in Dulbecco’s modified Eagle’s medium supplemented with 10% Fetal bovine serum and 1% penicillin/streptomycin. Cells were cultured in a humidified incubator with 5% CO_2_ at 37°C. Cells in the logarithmic growth phase were used for experiments, and the cell concentration was adjusted with PBS to 1.5 × 10^7^ cells/ml.

#### Tumor Xenograft in Nude Mice

This study was conducted in accordance with the principles of the Basel Declaration and guidelines developed by the Canadian Council on Animal Care. All of the protocols were approved by the Animal Care and Use Committee of the Shanghai University of TCM (Grant no. PZSHUTCM18111401). Four-week-old (weight: 9–13 g) male BALB/c nude mice were kept under pathogen-free conditions and a 12 h light-dark cycle. Mice were given free access to sterile food and water. HCT-116 cells (1.5 × 10^7^ cells/ml) were injected subcutaneously (s.c.) into the right forelimb near the axillary posterior scapular. Tumors became palpable 10 days after implantation. Animals with estimated mean tumor volumes of 80 to 200 mm^3^ were allocated to the control (*n* = 11), WCAF (*n* = 12), BEV (*n* = 12), and combination (*n* = 12) groups with almost equal mean tumor sizes and standard error. The experimenters were blinded to the details of an investigational treatment, data processing, and exclusionary decisions. The control group was treated with 0.2 ml of distilled water daily (by oral gavage) and 0.2 ml of physiological saline weekly intraperitoneally (i.p.). The WCAF group was treated daily with 0.368 g/ml WCAF by oral gavage, while the BEV group was treated weekly with 0.2 mg BEV (i.p.). The combination group received 0.368 g/ml of WCAF daily (by oral gavage) and 0.2 mg of BEV weekly (i.p.). Physiological saline (90%) was used as a diluent. After 6 weeks of treatment, when tumors reached ≥10% of their total body weight, all of the mice were euthanized by CO_2_ asphyxiation, and the tumors were excised. Tumors were then portioned for formalin fixation and paraffin embedment, and also for flash freezing and storage at −70°C.

#### Tumor Measurement and Observation

The maximum diameter (a) and width (b) of the tumors in each group were measured with a Vernier caliper every seven days. Tumor volume (V) was calculated according to the formula, V = ab^2^/2. Tumor growth inhibition by drug treatment was estimated according to the following formula: tumor inhibition rate (%) = (1—mean tumor weight in treatment group/mean tumor weight in control group) × 100%. After 6 weeks of treatment, the animals were euthanized by cervical spine dislocation, and the tumors were weighed using an electronic platform scale.

### Detection of *in situ* Cell Death by Terminal Deoxynucleotidyl Transferase-Mediated dUTP Nick-End Labeling

Apoptotic cells in paraffin sections of HCT-116 xenograft tumors were identified by the Terminal Deoxynucleotidyl Transferase-Mediated dUTP Nick-End Labeling (TUNEL) assay with the use of an apoptosis detection kit (*In Situ* Cell apoptosis Detection Kit, iPod; Boster, MK1020) according to the manufacturer’s instructions. The apoptotic cells were observed under a high power microscope. Five fields were randomly selected to count the number of apoptotic cells. Apoptosis rate = (TUNEL–positive cells/total cells) × 100%.

### Immunohistochemistry

Excised tumors were fixed, embedded, sectioned, and dewaxed. Antigen retrieval was then carried out using citric acid at high temperature. The sections were washed with PBS, incubated with an anti-CD31 antibody(1:400) anti-AKT1 antibody (1:25)and anti-FGF2 antibody (1:50)overnight, and they were then washed and incubated with a corresponding HRP-labelled secondary antibody at 37°C for 20–30 min. The sections were then rinsed with PBS, developed using DAB, and counterstained with hematoxylin and dehydrated. The CD31 protein expression in tissue sections was then evaluated using an optical inversion microscope (×400). Microvessels were evaluated by counting any positively stained endothelial cells or endothelial cell clusters in five areas with the highest vascular density. The mean number of microvessels in each field was regarded as the level of microvascular density (MVD). The expression levels of Protein kinase B(AKT1) and fibroblast growth factor 2(FGF-2) was semi quantitatively analyzed by image J.

### Immunoblot Analysis

The protein levels of Leptin-R, VEGFR-1, STAT3, p-STAT3, and BCL-2 after drug treatments were evaluated using Western immunoblotting. The total protein was extracted from the tumor tissues, and the total protein concentration was detected by the BCA Protein Concentration Assay Kit. Proteins were separated using SDS-PAGE and transferred to PVDF membranes. The membranes were blocked in 5% bovine serum albumin (BSA) for 2 h and then washed with TBST three times, each for 5 min on a shaker. The primary antibodies used were Leptin-R (Abcam 1:2000), STAT3 (CST 1:2,000), p-STAT3 (CST 1:1,000), BCL-2 (Abcam 1:2,000), VEGFR-1 (Abcam 1:2,000), and β-actin (Abcam 1:2,000). After incubation overnight at 4°C with primary antibodies, the membranes were washed with TBST and then incubated with one of the following secondary antibodies at room temperature for 1 h: HRP-labeled goat anti-rabbit secondary antibody (Jackson 1: 2,000) or HRP-labeled goat anti-mouse secondary antibody (Jackson 1:2,000). The immunoblots were then visualized using enhanced chemiluminescence (ECL) reagents and exposed to X-ray film. The membranes were subsequently re-probed with anti-β-actin antibody. Western blots were performed according to the manufacturer’s instructions with standards run in triplicate. The gray values of the immunoblots were semi-quantitatively determined by image analysis.

### Real-Time PCR

At the end of treatment, the mice were euthanized, and tumor samples were frozen in liquid nitrogen and stored at −80°C. The gene levels of Leptin and *VEGF-A* after drug treatments were evaluated using RT-PCR. RNA was isolated using TRIzol, and then the concentration and RNA quality were measured. Based on the sequence of the target gene in the Gene Bank database, the primers were designed using Primer Express Software v2.0 and synthesized by the Beijing Genomics institution. The sequences of the primers used for PCR were Leptin GGA​TGA​CAC​CAA​AAC​CCT​CA (FP), Leptin CAG​GAA​TGA​AGT​CCA​AGC​CA (RP), VEGF-A ATC​TTC​AAG​CCG​TCC​TGT​GT (FP), VEGF-A AGG​TTT​GAT​CCG​CAT​GAT​CT (RP), mGAPDH GGC​AAA​TTC​AAC​GGC​ACA​GT (FP), and mGAPDH ACG​ACA​TAC​TCA​GCA​CCG​GC (RP). RT-PCRs were performed in triplicate for each sample.

### Network Pharmacology

The compounds of the herbs identified in WCAF were obtained from the TCM systems pharmacology database and analysis platform (TCMSP, http://lsp.nwu.edu.cn/browse.php?qc=herbs). ADME (absorption, distribution, metabolism, and excretion) parameters were used to screen out the possible drug molecules, and ADME data came from tcmsp database. The common screening crite-ria are oral bioavailability (OB) ≥30% and drug-likeness (DL) ≥0.18. The TCMSP and BATMAN (http://bionet.ncpsb.org/batman-tcm/) databases were selected to predict the target proteins for WCAF. bioactive ingredients.

In order to search the genes related to clone cancer in the Comparative Toxicogenomics Database (CTD) (http://ctdbase.org/), the “clone cancer” “Colonic Neoplasms” were used as key words, and ranking was conducted based on the inference score (Barabasi and Albert, 1999; Hua and Shoudan, 2009).Target proteins with scores >20,which were also target proteins predicted in the previous step, were identified in order to narrow the range of target proteins.Inference scores were calculated using the logarithmic transformation products of two common neighborhood statistics and then used to evaluate the functional correlation.between proteins in protein-protein interaction (PPI) networks.

Go (Gene ontology) function network analysis of target protein was performed using Cytoscape plugins cluego + cluepedia (Bindea et al., 2009), the significance threshold is set as p.adjust ≤0.01. Each cluster selects more than 10 genes, and uses Cytoscape as the target function network diagram.

KEGG (Encyclopedia of Genes and Genomes) signaling pathway enrichment analysis of the target protein was carried out based on the R-package clusterprofiler The significance threshold was set as *p* ≤ 0.01, and the bubble chart was made for visualization.

We used string (version: 10.0, http://www.string-db.org/）database to analyze the PPI of mRNA. Select required confidence (combined score) > 0.4 as the threshold value of PPI relationship. Based on herbal medicine component target protein pathway data generated by previous steps, screening WCAF and cancer immune related pathway to build pharmacology network. pharmacological network was constructed by using Cytoscape.

### Statistical Analysis

The collected data were analyzed using SPSS v.17 (SPSS Inc., Chicago, IL, United States). In this study, the data were continuously measured using a 2 × 2 factorial design. If there were interactions, we analyzed individual effects. If there were no interactions, we analyzed the main effect. Data with normal distribution are presented as mean ± standard deviation (mean ± SD), using one-way analysis of variance (ANOVA) followed by a *post-hoc* least significant difference (LSD) to compare the effect of the mean difference. Prior to analyses, variables were tested for normality using the Shapiro-Wilk test for homogeneity of variances with Levene’s test. Data without normal distribution are presented as mean ± (lower quartile – upper quartile), and the mean difference in the effects was compared using the Kruskal-Wallis method. *p* < 0.05 was considered to be statistically significant.

## Results

### Mice Body Weight

**FIGURE 2:**
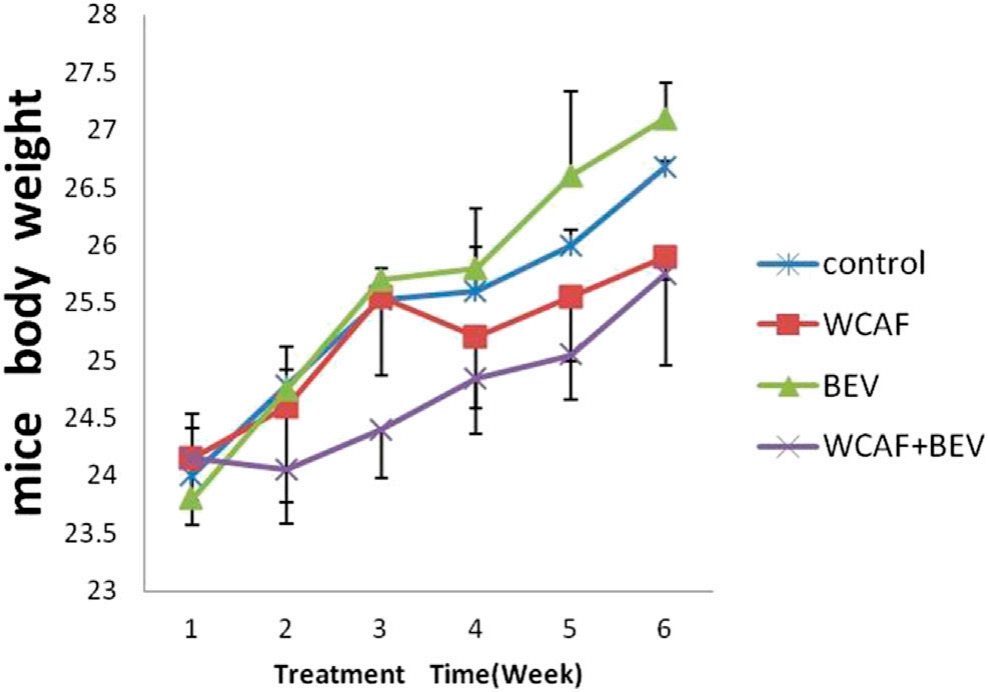
mice body weight curve.

Mice body weight were monitored every 7 days. Mice body weight of treated groups showed a growth pattern that did not deviate significantly from those of the control group (Figure 2). There was a slight decline in the body weight of mice group treated with WCAF + BEV from week 1 to week 2; however, their body weight increased gradually from Week 2 to week 6. Those treated with WCAF alone demonstrated a slight decline in body weight from week 3 to week 4, then increased gradually from Week 4 to week 6.

### Effect of Weichang’an Formula on the Growth of Human Colon Cancer HCT-116 Xenografts in Nude Mice

**FIGURE 3:**
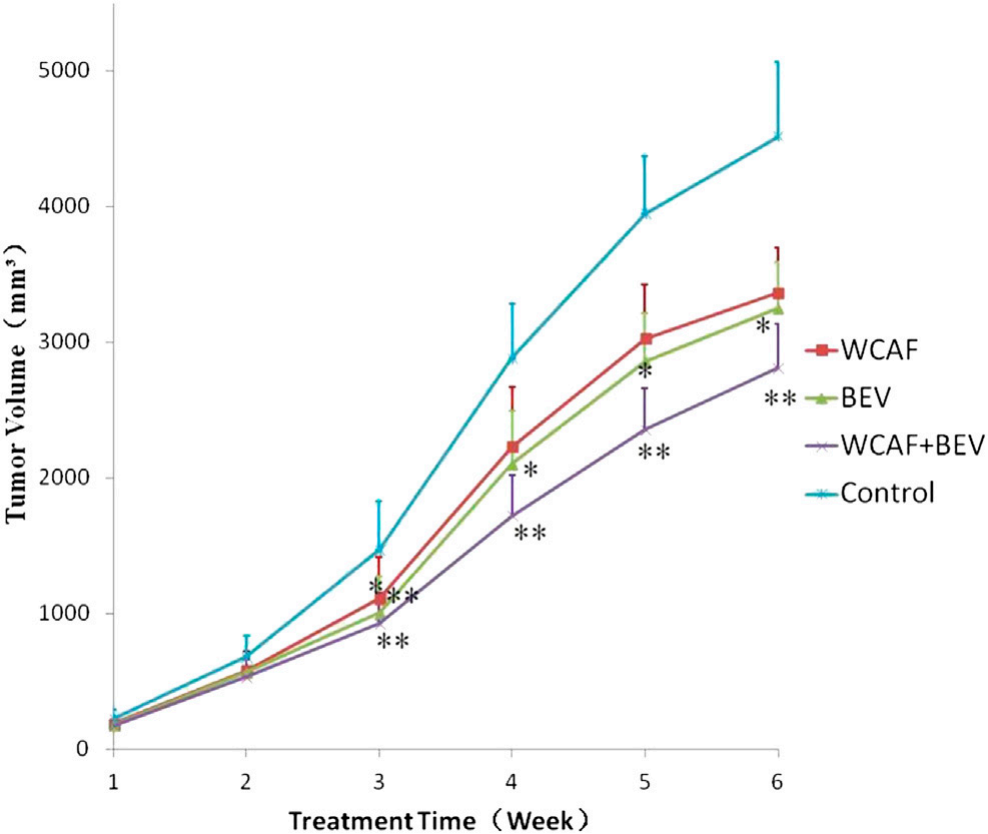
Tumor growth curves based on different treatments.^*^ *p* < 0.05 vs. control group, ^**^ *p* < 0.01 vs. control group.

Implanted tumors were monitored by gross observation and measurement of tumor volume every 7 days. The growth curves of tumor-bearing mice in each of the four groups showed that the tumor volume increased with time and that the tumor volumes were as follows: Control > WCAF > BEV > WCAF + BEV (Figure 3).

**FIGURE 4:**
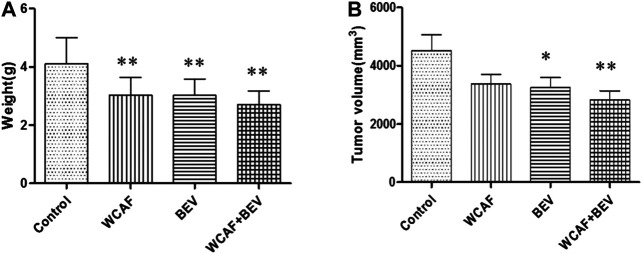
Tumor weight, volume, based on drug treatment at week 6. *p < 0.05 vs. control group, ^**^ *p* < 0.01 vs. control group.

At the end of treatment, the tumor volume and tumor weight inhibition rate were calculated as follows: tumor volume inhibition rate = (control group tumor volume – drug group tumor volume)/control group tumor volume × 100%, and tumor weight inhibition rate = (control group tumor weight–drug group tumor weight)/control group tumor mass × 100%. The number of mice included in the statistical analysis was seven in the control group, 11 in the WCAF group, 11 in the BEV group, and 12 in the combination group. Four mice in the control group and one in the WCAF and BEV groups were excluded from the analysis due to tumor ulceration. The average volume and average weight of tumors in the WCAF, BEV, and combination groups were lower than those in the control group. The results showed enhanced tumor control with the combined application of TCM (WCAF) and western medicine (BEV). At week 6, The mean tumor volume in the control group was significantly different from the mean tumor volume in both the BEV (*p* < 0.05) and WCAF + BEV groups (*p* < 0.01),. The tumor weights of the BEV, WCAF, and the combination group were all significantly lower than that of the control group (*p* < 0.01). (Figures 4) The tumor inhibition rate of each group was 25.48% in the WCAF group, 28.06% in the BEV group, and 37.77% in the combination group (Table 1).

**TABLE 1:** Tumor weight, volume, and inhibition rate based on drug treatment (mean ± SD).

Group	*n*	Weight (g)	Tumor volume (mm³)	Tumor inhibition (%)
Control	7	4.11 ± 0.89	4,518.29 ± 544.30	—
WCAF	11	3.04 ± 0.60^a^	3,367.82 ± 327.69	25.48
BEV	11	3.03 ± 0.56^a^	3,251.82 ± 344.82	28.06
WCAF + BEV	12	2.71 ± 0.46^a^	2,815.00 ± 327.19^b^	37.77

a *p* < 0.01 vs. control group.

b *p < 0.05* vs. control group.

### Effect of Weichang’an Formula on Apoptosis and Angiogenesis of Human Colon Carcinoma HCT-116 Xenografts

**FIGURE 5:**
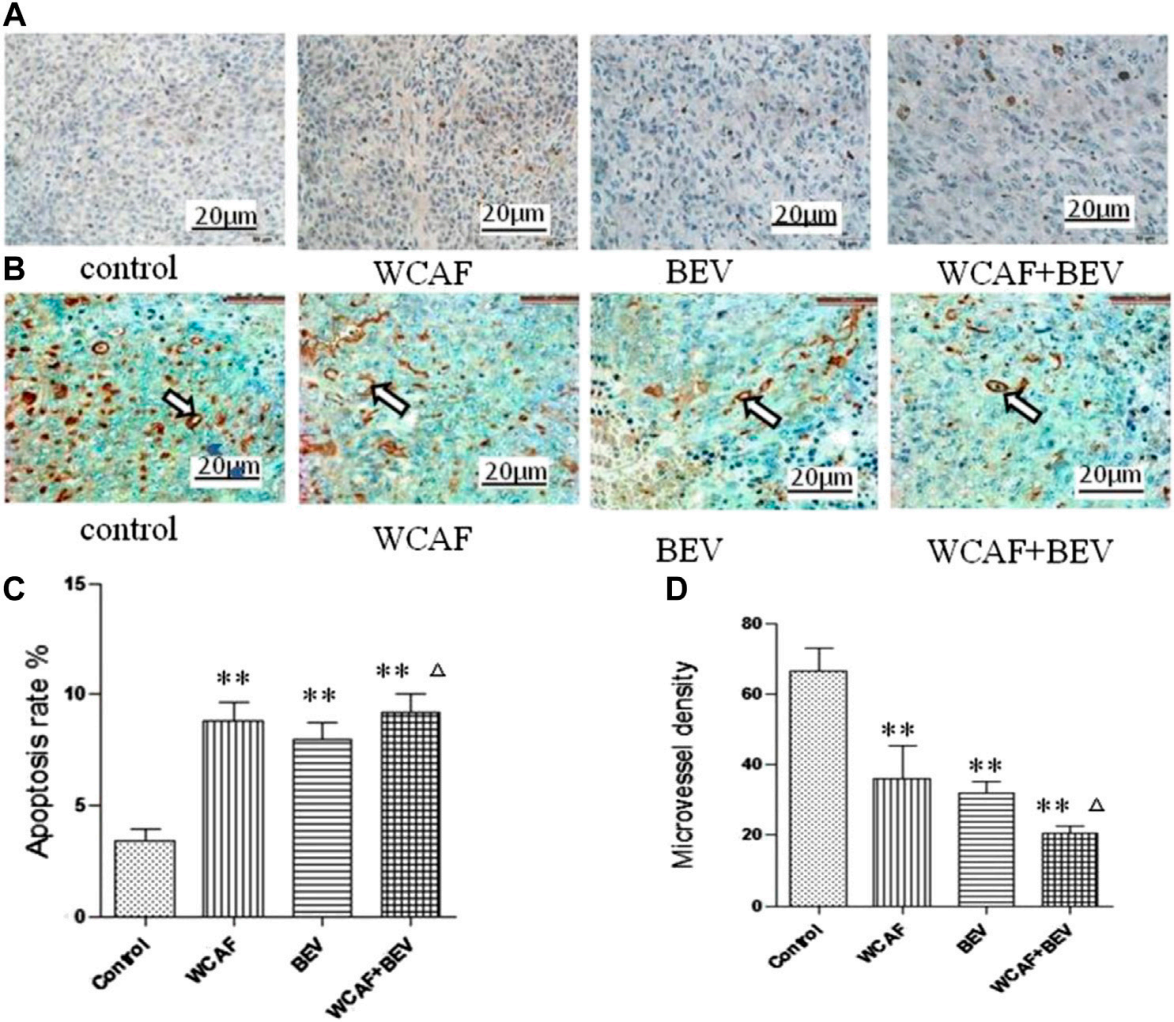
Effect of WCAF on apoptosis and angiogenesis in colon carcinoma. **(A,C)** Tumor apoptosis was assessed by TUNEL assay (×400), ^**^ *p* < 0.01 vs. control group. ^△^ *P<* 0.05 vs BEV group **(B,D)** Expression of CD31 protein in four subcutaneous xenografts in nude mice (immunohistochemical staining, ×400), ^**^ *p* < 0.01 vs. control group. ^△^ *P<* 0.05 vs BEV group.

TUNEL staining was used to detect the level of apoptosis in the control (3.4 ± 0.55%), WACF (8.8 ± 0.84%), BEV (8 ± 0.71%), and combination (9.2 ± 0.84%) groups. Statistical analysis showed that there was significantly more apoptosis in the WCAF, BEV, and combination groups in comparison to the control group (*p* < 0.01). The difference between the combination group and BEV alone was statistically significant (*p <* 0.05) (Figures 5A,C).

CD31 protein expression in tumor tissues was examined by Immunohistochemistry (IHC). CD31 protein positive expression was mainly located in the cytoplasm of vascular endothelial cells. CD31-positive vascular endothelial cells were counted to calculate the MVD. The MVD in each group was 66.4 ± 6.80 (control group), 36 ± 9.49 (WCAF group), 31.8 ± 3.35 (BEV group), and 20.4 ± 2.30 (WCAF + BEV group). The MVD in the WCAF, BEV, and WCAF + BEV groups was significantly lower than the MVD in the control group (*p <* 0.01). The difference on MVD between the combination group and BEV alone was statistically significant (*p <* 0.05) (Figures 5B,D).

### Effect of Weichang’an Formula on Leptin, Leptin-R, STAT3, and p-STAT3 Expression in HCT-116 Xenografts in Nude Mice

**FIGURE 6:**
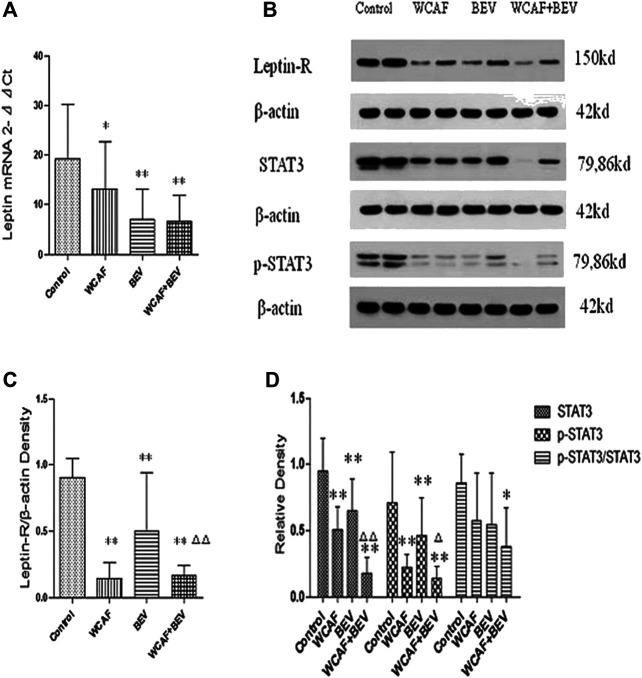
Effect of WCAF on Leptin, Leptin-R, STAT3, and p-STAT3 expression. **(A)** RT-PCR analysis of Leptin mRNA expression. Data are presented as mean. *p < 0.05, ^△^ *p* < 0.01 compared with the control group. **(B)** Western blot analysis of the protein expression of LeptinR-1, STAT3, and p-STAT3. **(C)** Quantification of band intensities of Leptin-R. ^**^ *p* < 0.01 vs. control group. ^△△^ *p* < 0.01 vs. BEV group. **(D)** Quantification of band intensities of STAT3, p-STAT3, and p-STAT3/STAT3. *p < 0.05 vs. control group, ^**^ *p* < 0.01vs. control group. ^△^ *P<* 0.05 vs BEV group.^△△^ *p* < 0.01 vs. BEV group.

RT-PCR was used to detect the transcription level of Leptin in the implanted tumors. All of the values were first compared to the internal parameter of ΔCt, and then the first sample in the BEV group was used to do the relative content = 2^−ΔΔCt^ analysis. The relative content of Leptin mRNA in each group was as follows: 19.14 ± 11.12 (control group), 13.22 ± 9.52 (WCAF group), 7.13 ± 5.97 (BEV group), and 6.64 ± 5.36 (combination group). Leptin mRNA levels were significantly lower in the WCAF, BEV, and combination groups than in the control group (*p < 0.05*). There was no statistically difference on Leptin mRNA expression between the combination group and BEV alone (Figures 6A).

The expression of Leptin-R, STAT3, and p-STAT3 in tumors was detected by Western blot analysis. Tumor Leptin-R levels were lower in the WCAF, BEV, and combination groups than in the control group. The ratio of Leptin-R to β-actin in the control group was 0.90 ± 0.12, 0.14 ± 0.10 in the WCAF group, 0.50 ± 0.38 in the BEV group, and 0.16 ± 0.07 in the combination group. Therefore, the ratio of Leptin-R/β-actin was significantly lower in the BEV, WCAF, and combination groups in comparison to the control group (*p* < 0.01). The ratio of Leptin-R/β-actin between the combination group and BEV alone achieved statistically significant difference (*p* < 0.01) (Figures 6B,C).

STAT3 and p-STAT3 levels in tumors from nude mice in the WCAF, BEV, and combination groups were significantly lower than the levels in the control group. The ratio of STAT3 to β-actin was 0.95 ± 0.25 in the control group, 0.51 ± 0.17 in the WCAF group, 0.65 ± 0.24 in the BEV group, and 0.18 ± 0.12 in the combination group. Therefore, the ratio of STAT3 to β-actin was significantly lower in the WCAF, BEV, and combination groups than in the control group (*p* < 0.01). The ratio of STAT3/β-actin between the combination group and BEV alone achieved statistically significant difference (*p* < 0.01).The ratio of p-STAT3 to β-actin was 0.71 ± 0.38 in the control group, 0.22 ± 0.10 in the WCAF group, 0.46 ± 0.29 in the BEV group, and 0.14 ± 0.09 in the combination group. Therefore, the ratio of p-STAT3 to β-actin was significantly lower in the WCAF, BEV, and combination groups than in the control group (*p < 0.01*). There was statistically difference on p-STAT3 protein expression between the combination group and BEV alone (*p < 0.05*).The ratio of p-STAT3/STAT3 was 0.86 ± 0.22 in the control group, 0.58 ± 0.35 in the WCAF group, 0.55 ± 0.39 in the BEV group, and 0.39 ± 0.29 in the combination group. Therefore, the ratio of p-STAT3/STAT3 was significantly lower in the combination group than in the control group (*p* < 0.05) (Figures 6B,D).

### Effect of Weichang’an Formula on the Expression of VEGF-A, VEGFR-1, and BCL-2 in HCT-116 Xenografts in Nude Mice

**FIGURE 7:**
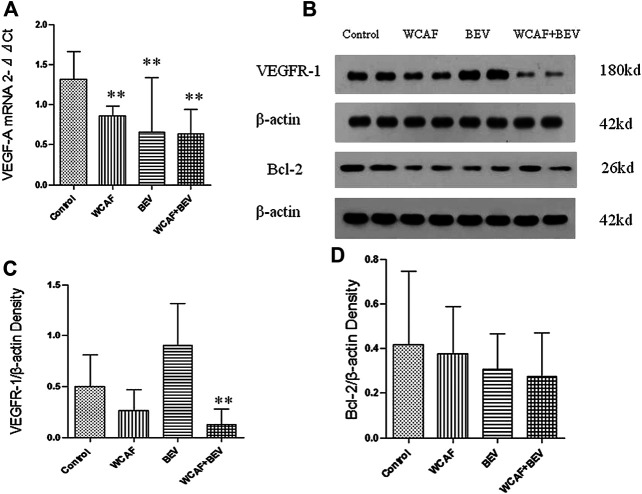
Effect of WCAF on the expression of VEGF-A, VEGFR-1, and BCL-2. **(A)** RT-PCR analysis of *VEGF-A* mRNA expression. ^**^ *p* < 0.01 vs. control group. **(B)** Western blot analysis of the protein expression of VEGFR-1 and BCL-2. **(C)** Quantification of band intensities of VEGFR-1 expression.^**^ *p* < 0.01 vs. control group. ^△△^ *p* < 0.01 vs. BEV group.**(D)** Quantification of band intensities of BCL-2 expression. ^**^ *p* < 0.01 vs. control group.

RT-PCR method was used to detect the gene transcription level of *VEGF-A* in subcutaneous xenografts of nude mice. All of the values were first compared to the internal parameter of ΔCt, and then the first sample in the BEV group was used to do the relative content = 2^−ΔΔCt^ analysis. The relative level of *VEGF-A* mRNA in each group was as follows: 1.32 ± 0.34 (control group), 0.86 ± 0.12 (WCAF group), 0.66 ± 0.68 (BEV group), and 0.64 ± 0.31 (combination group). The expression of *VEGF-A* mRNA was significantly lower in the WCAF, BEV, and WCAF + BEV groups than in the control group (*p* < 0.01). But there was no statistically difference on *VEGF-A* mRNA expression between the combination group and BEV alone (Figures 7A).

The expression of *BCL-2* and *VEGFR-1* in the implanted tumors was detected by Western blot analysis. VEGFR-1 was significantly lower in the WCAF and the combination group in comparison to the control group. The ratio of *VEGFR-1* to *β-ACTIN* in the control group was 0.50 ± 0.27, 0.26 ± 0.18 in the WCAF group, 0.91 ± 0.35 in the BEV group, and 0.12 ± 0.14 in the combination group. The results showed that the ratio of *VEGFR-1* to *β-ACTIN* in the combination group was significantly lower than that in the control group (*p* < 0.01). The ratio of VEGFR-1/β-actin between the combination group and BEV alone achieved statistically significant difference (*p* < 0.01) (Figures 7B,C).

The expression of the BCL-2 protein was lower in the subcutaneous xenografts of the nude mice in the WCAF, BEV, and combination groups than in the control group. The ratio of BCL-2 to β-ACTIN was 0.42 ± 0.30 in the control group, 0.37 ± 0.18 in the WCAF group, 0.31 ± 0.14 in the BEV group, and 0.28 ± 0.05 in the combination group. While the differences were not statistically significant (*p* > 0.05), there was a decreasing trend in comparison to the control group (Figures 7B,D).

### Gene Ontology and KEGG Pathway Analyses

**FIGURE 8:**
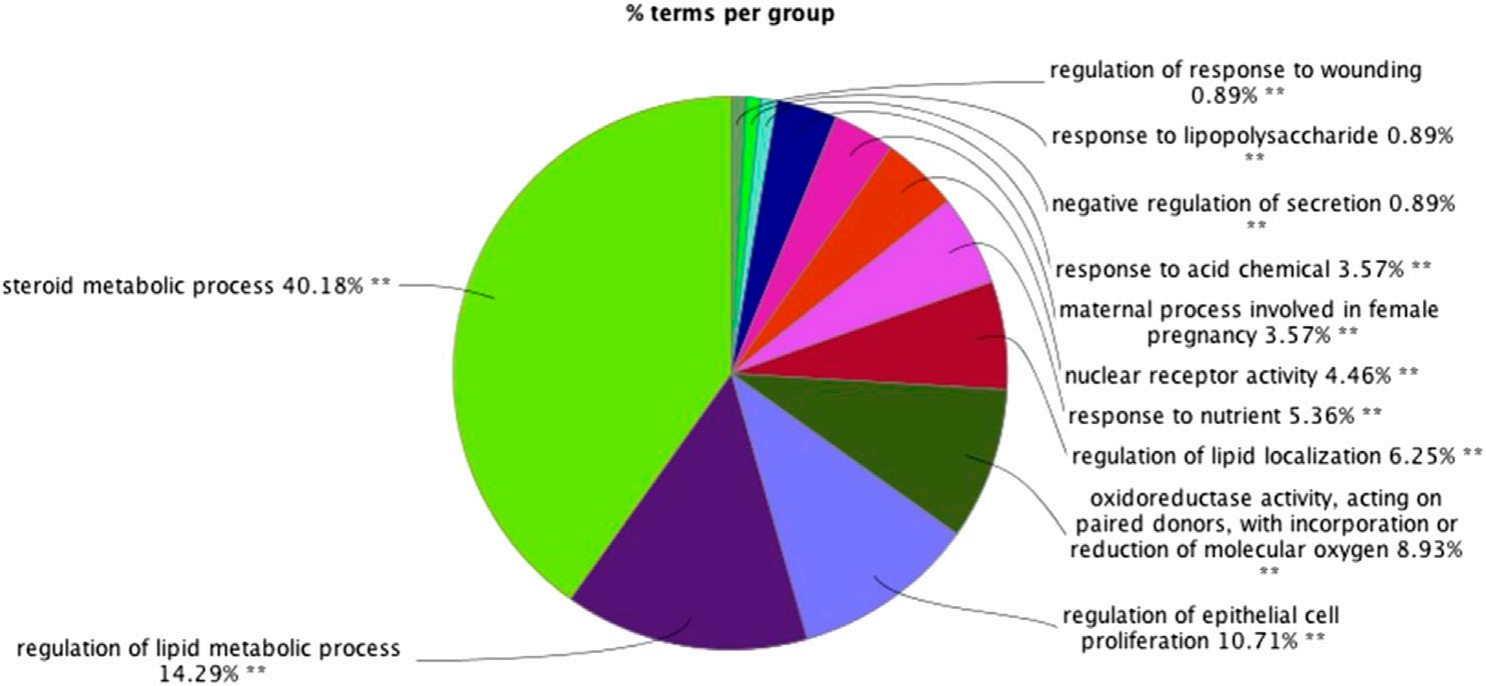
The results of gene ontology (GO) functional analyses: **(A)** histogram; **(B)** pie chart.∗∗*p* < 0.01*p < 0.05.

**FIGURE 9:**
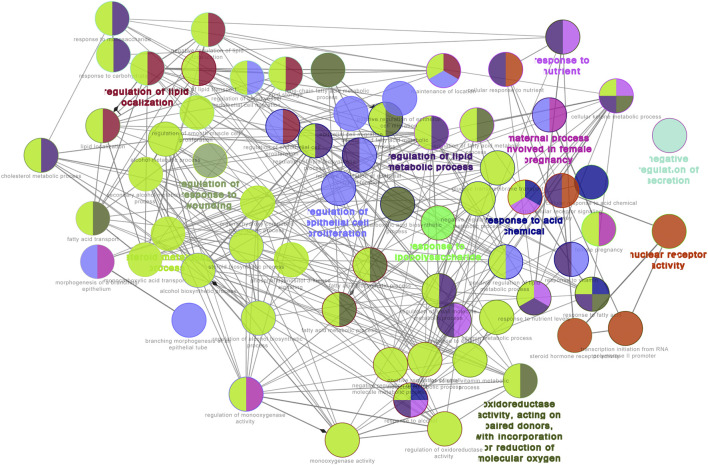
GO functional network for 73 target proteins. Nodes:GO terms; bigger nodes indicated smaller *p* values;the two-point line represents the correlation between functions, and the larger the kappa coefficient, the thicker the line.

**FIGURE 10:**
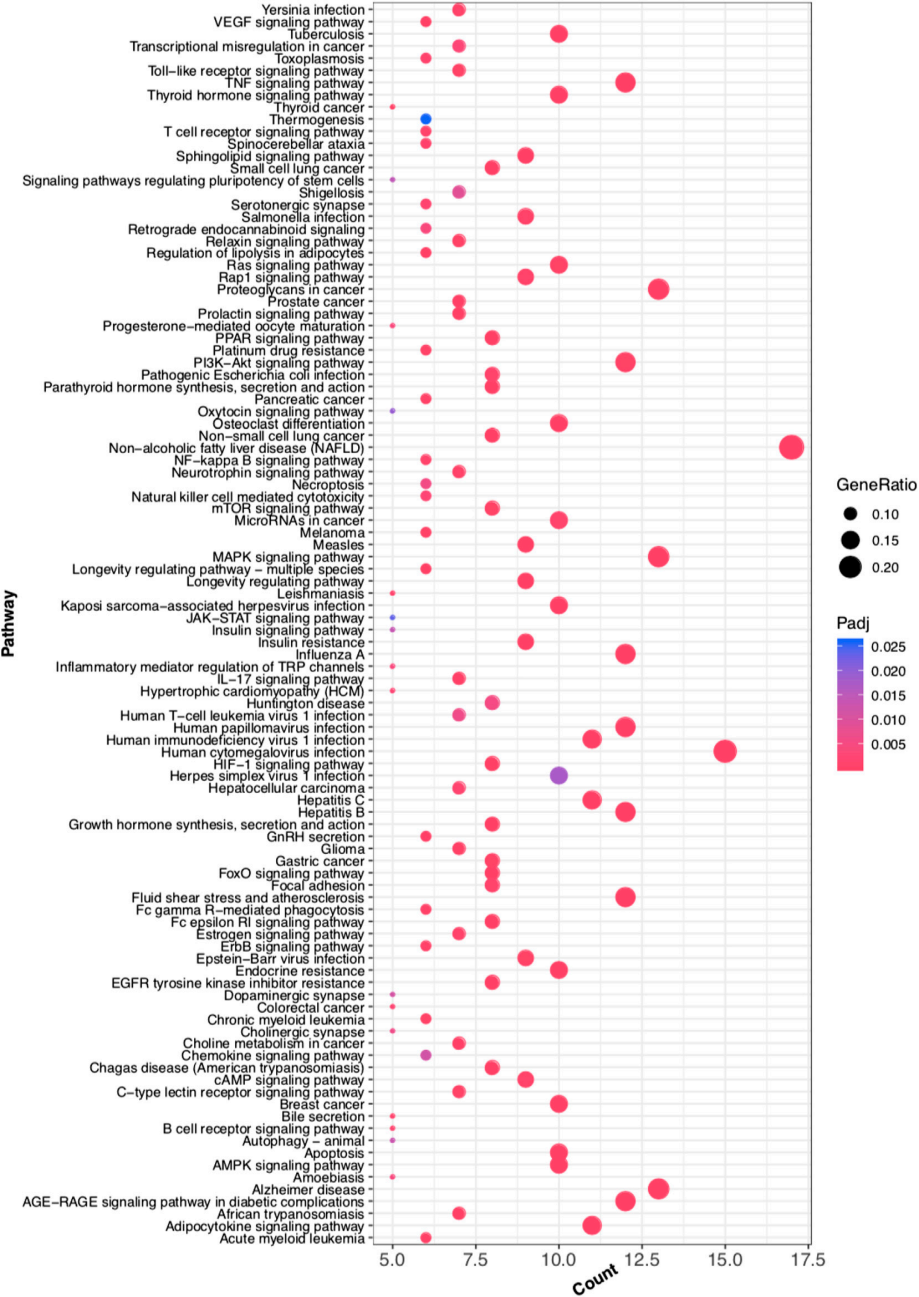
Bubble Diagram of target protein pathway enrichment. Te longitudinal axis: the pathway name; the transverse axis: the number of enriched genes; the size of the dot represents the proportion of the number of enriched genes to the total number of genes, and the larger the proportion is, the larger the dot is; the redder the dot color, the more significant the *p* value.

GO functional analysis showed that a total of 67 GO function items were enriched, and that the GO function was divided into 12 categories based on the kappa coefficient (Figure 8). The GO functional net-work indicated that the 73 target proteins were enriched in 67 biological processes (Figure 9). The cross target gene was enriched by cluster profiler, and there were 134 significant pathways. Bubble chart shows pathways with *p* < 0.01 and number of enriched genes ≥0.05 (Figure 10). The enriched pathways include colorectal cancer pathway, human immunodeficiency virus 1 infection pathway, nonalcoholic fatty liver, proteoglycan in cancer and other pathways; immune related pathways include JAK-STAT signaling pathway, apoptosis, PI3K Akt signaling pathway and MAPK signaling pathway NF kappa B signaling pathway, T cell receptor signaling pathway and B cell receptor signaling pathway

### Protein-Protein Interaction Network Analysis

**FIGURE 11:**
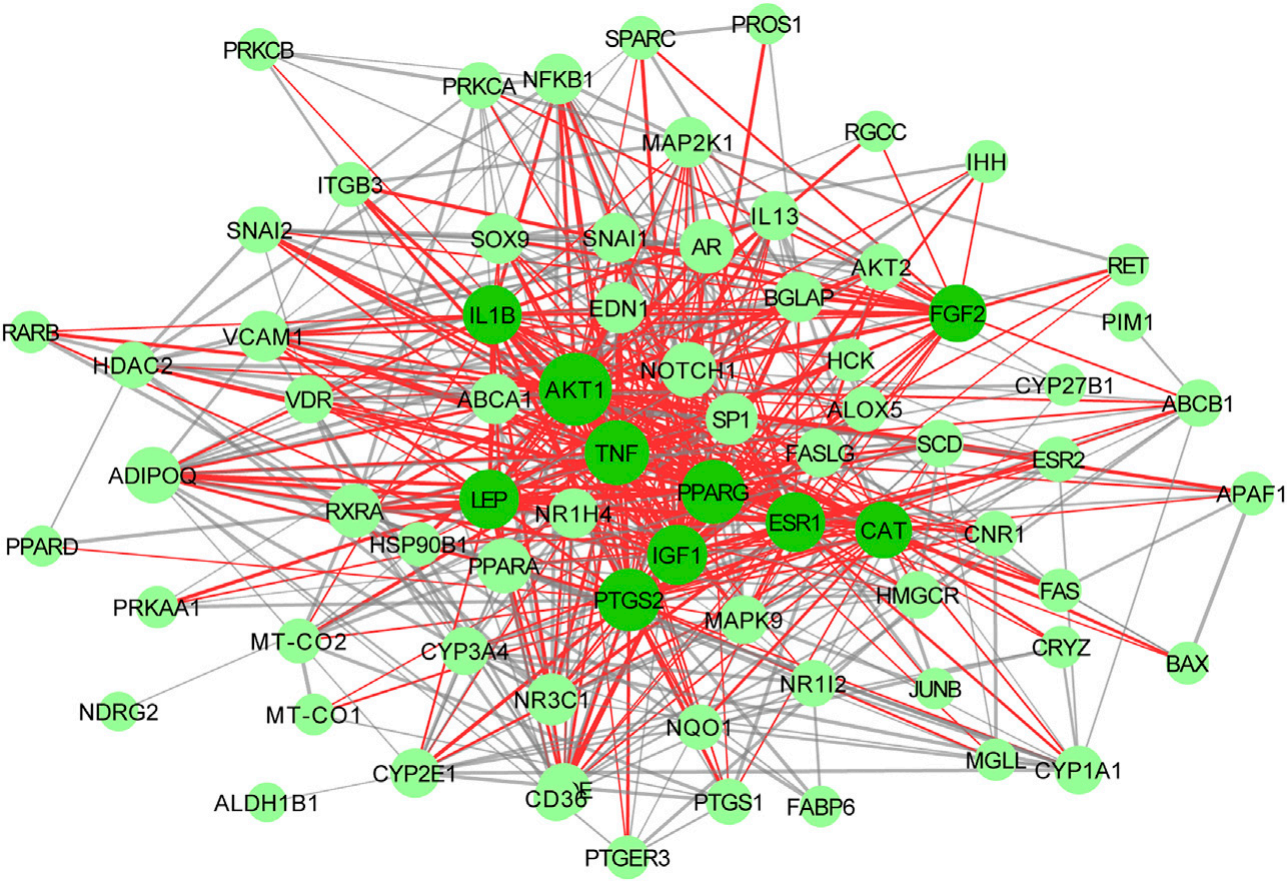
Interactive PPI network of WCAF and colorectal cancer. with 71 nodes and 551 edges: nodes indicate target proteins or genes; edges represent correlations between targets; the size of the nodes indicates the value of degree.

74 proteins and 551 relation pairs were found in the PPI network (Figure 11). The top 10 proteins with higher degrees were AKT1 (degree = 50), TNF (degree = 35), PPARG (degree = 39), PTGS2 (degree = 37), IGF1 (degree = 33), LEP (degree = 32), ESR1 (degree = 32), IL1B(degree = 31), FGF2(degree = 29), and CAT (degree = 28) (Table 2)

**TABLE 2:** Top 10 genes of WCAF-Colorectal cancer PPI network according to degree.

Gene	Degree	Betweenness	Closeness
AKT1	53.0	915.56555	0.7765958
TNF	40.0	314.9559	0.682243
PPARG	39.0	366.88446	0.6636364
PTGS2	37.0	323.56192	0.6636364
IGF1	33.0	219.30272	0.6347826
LEP	32.0	150.87827	0.6403509
ESR1	32.0	195.33363	0.6347826
IL1B	31.0	129.34435	0.61864406
FGF2	29.0	133.4995	0.60833335
CAT	28.0	183.36874	0.60330576

### Construction of Pharmacological Network

**FIGURE 12:**
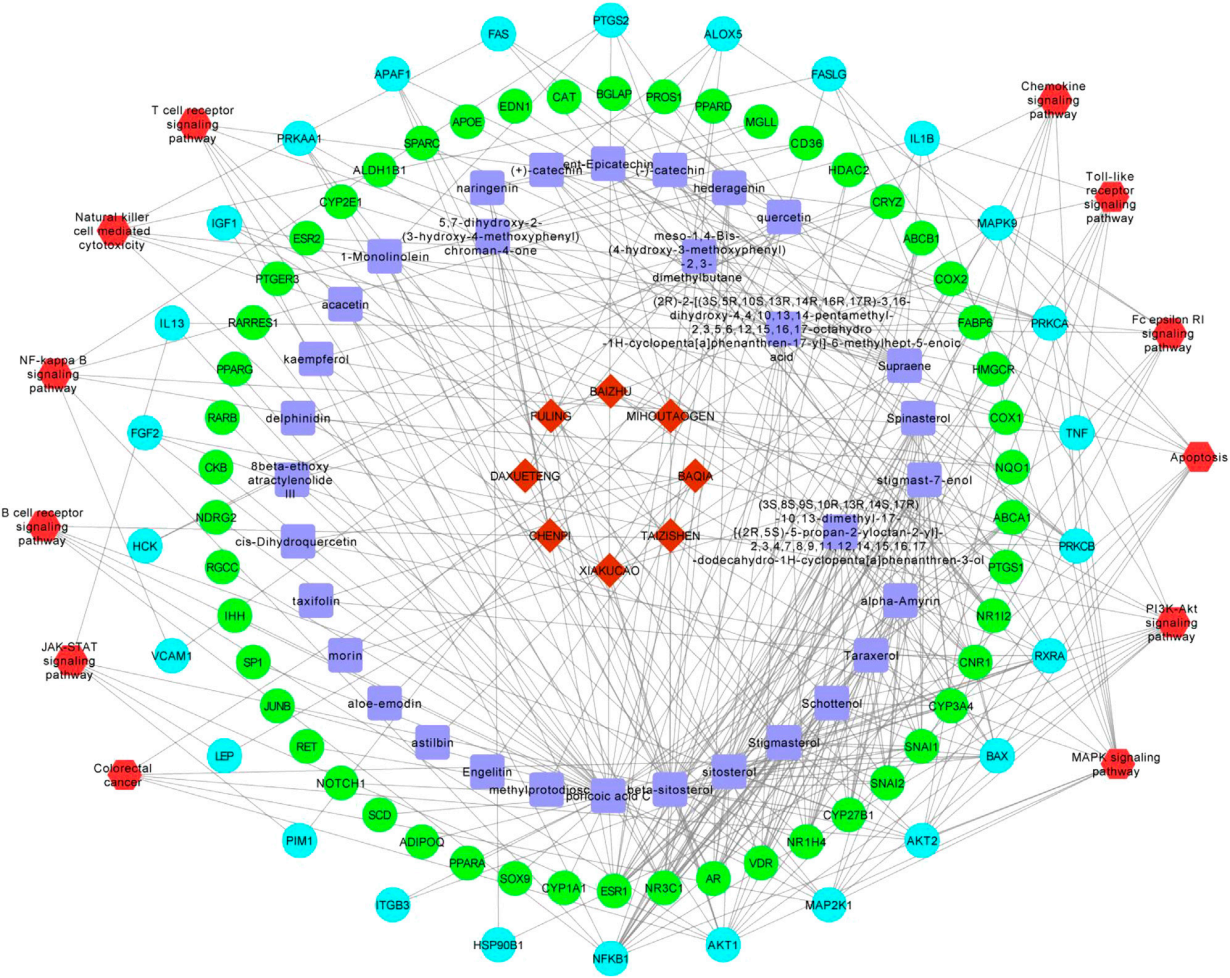
The pharmacological network. Red hombus:herbal medicine; blue square:components; light blue dots: pathway related targets; green dot: non pathway related targets;red hexagon:pathways.

Based on the herbal medicine-component-target protein pathway, the network pharmacology network was constructed by screening cancer immune related pathways. Finally, 12 related paths are selected to build the network as shown in Figure 12. There are 128 nodes and 372 relation pairs in the network, including eight herbal nodes, 33 chemical composition nodes (including beta sitosterol and sitosterol), 76 target protein nodes, and 26 pathway related target protein nodes.

### Effect of Weichang’an Formula on AKT1 Expression of Human Colon Carcinoma HCT-116 Xenografts

**FIGURE 13:**
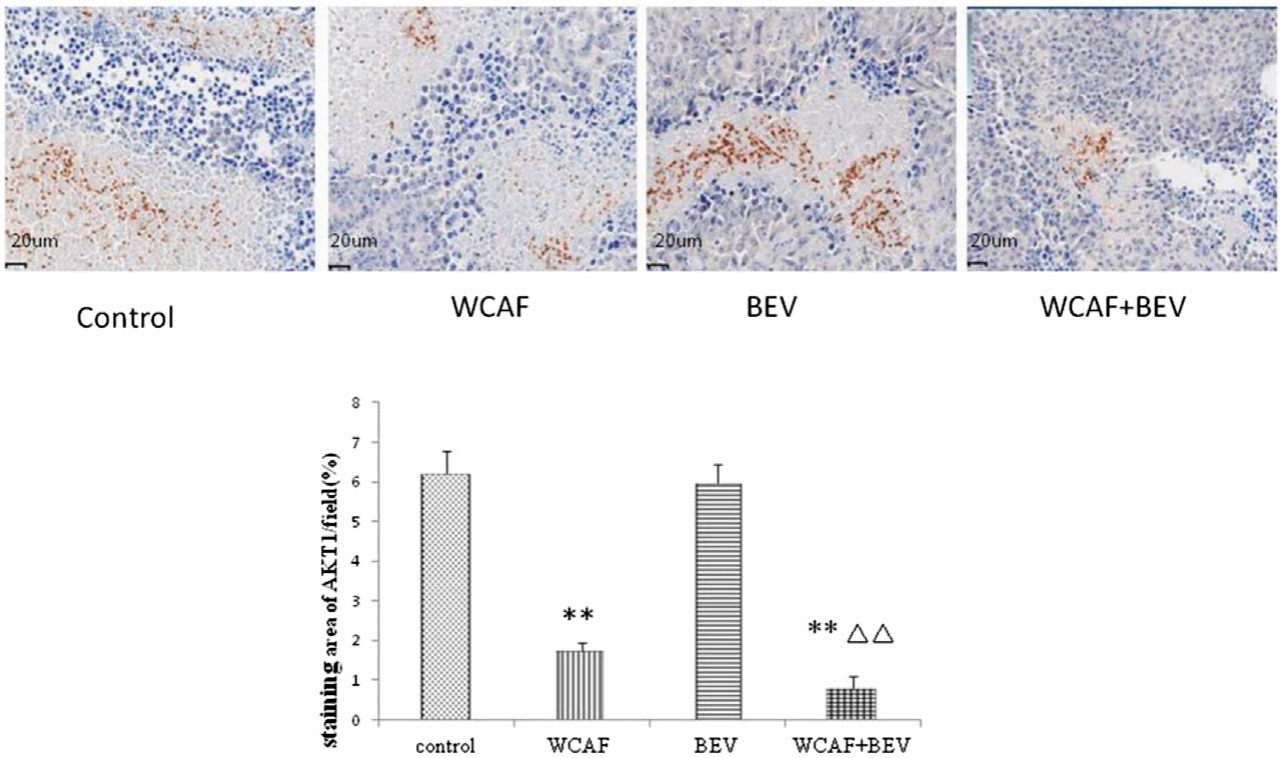
Effect of WCAF on AKT1 expression in colon carcinoma. **p < 0.01 vs. control group. △△*p* < 0.05 vs BEV group.

According to the prediction of the network pharmacology molecular target of WCAF for regulating the immunity of colorectal cancer, we chose AKT1 for verification.AKT1 protein expression in tumor tissues was examined by IHC. AKT1 is mainly expressed in necrotic tumor tissue and located in nucleus. The staining area of AKT1/field (%) in each group was 6.22 ± 0.57 (control group), 1.74 ± 0.21 (WCAF group), 5.95 ± 0.50 (BEV group), and 0.81 ± 0.30 (WCAF + BEV group). The AKT1 protein expression in the WCAF and WCAF + BEV groups was significantly lower than the that in the control group (*p <* 0.01). The difference on AKT1 protein expression between the combination group and BEV alone was statistically significant (*p <* 0.01) (Figure 13).

**FIGURE 14:**
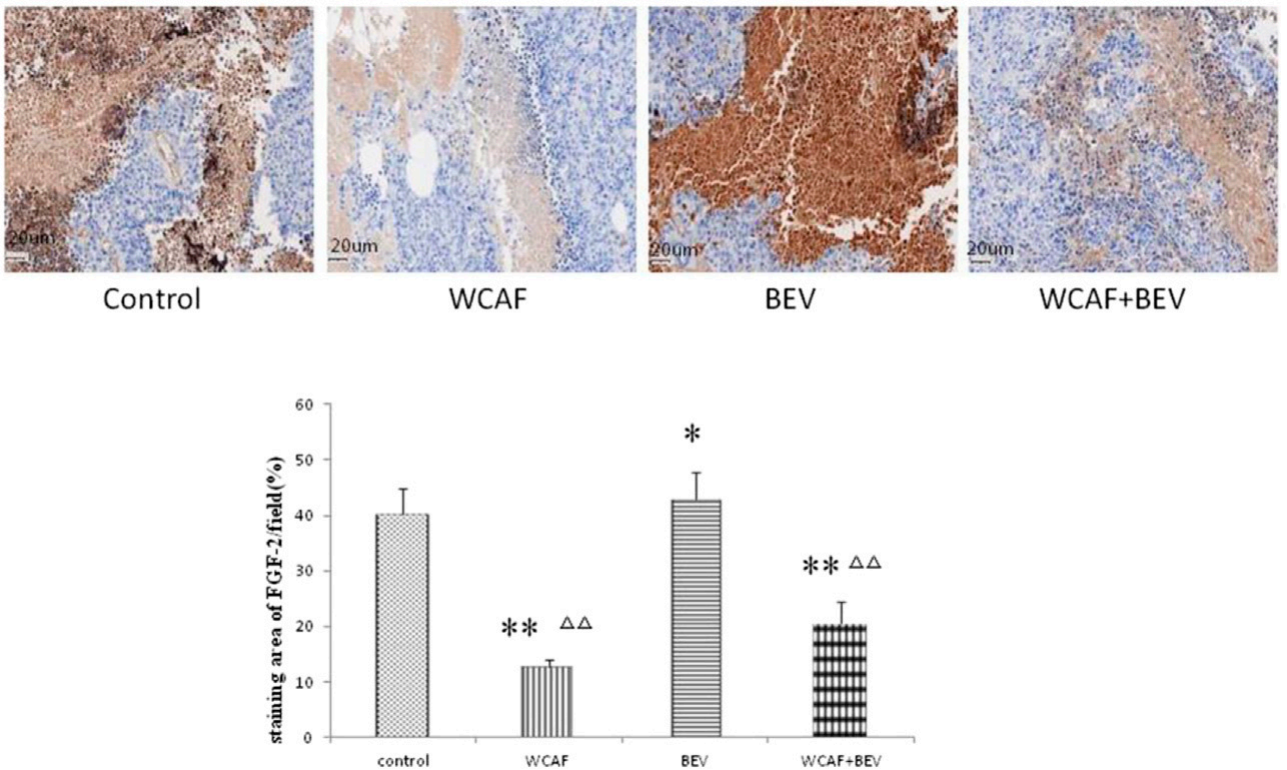
Effect of WCAF on FGF-2 expression in colon carcinoma. ^**^ *p* < 0.01 vs. control group. ^△△^ *P<* 0.05 vs BEV group.

### Effect of Weichang’an Formula on FGF-2 Expression of Human Colon Carcinoma HCT-116 Xenografts

According to the prediction of the top 10 network pharmacology molecular target of WCAF for regulating the immunity of colorectal cancer, we chose FGF-2 for verification. FGF-2 protein expression in tumor tissues was examined by IHC. The staining area of FGF-2/field (%) in each group was 40.26 ± 4.75 (control group), 12.71 ± 1.23 (WCAF group), 42.83 ± 4.95 (BEV group), and 20.60 ± 3.94 (WCAF + BEV group). The FGF-2 protein expression in the WCAF and WCAF + BEV groups was significantly lower than the that in the control group (*p <* 0.01). The FGF-2 protein expression in the BEV group was higher than the that in the control group (*p <* 0.05). The difference on FGF-2 protein expression between the combination group and BEV alone was statistically significant (*p <* 0.01) (Figure 14).

## Discussion

TCM plays an active role in the treatment of colorectal cancer. It can be used to supplement surgical treatment, radiation therapy, chemotherapy, immunotherapy, targeted therapy, and other western medicine treatments of colorectal cancer. It can also be used as a monotherapy treatment option for patients with colorectal cancer. TCM has several treatment advantages, including stabilizing the condition, improving the immune function and the quality of life of patients, and extending patient survival. WCAF was developed by Professor Qiu Jiaxin, a renowned Chinese medicine practitioner in Shanghai, and his team based on their vast clinical and pharmaceutical research experience. WCAF is mainly composed of heat-clearing and toxicity-removing herbs and the active ingredient sijunzifang, which can nourish the spleen. Sijunzifang can inhibit SGC-7901 cell proliferation by G1/G0 phase arrest and induce apoptosis by upregulating BAX and downregulating BCL-2 (Qian et al., 2016). *Radix Actinidiae Chinensis* is one of the heat-clearing and toxicity-removing herbs that can reduce HCC cell viability, and which has been shown to have anti-tumor activity (He et al., 2017). The combination of WCAF and 5-FU significantly inhibited colon tumor growth and hepatic metastases (Tao et al., 2015).In this study, our results indicate that WCAF can inhibit the tumor growth of human colon cancer HCT-116 nude mouse subcutaneous xenografts. WCAF in combination with the anti-angiogenic targeted agent BEV had a partial additive inhibitory effect on the growth of human colon cancer HCT-116 xenografts in nude mice. In this study, we found that on day 52 after tumor implantation, four of the 11 mice in the control group developed ulceration, suggesting that we should shorten the treatment window in the future.

In 1971, Judah Folkman first proposed that tumor growth and metastasis required new blood vessel formation (Folkman, 1971). Tumor cells and blood vessels can form a highly integrated ecosystem that allows tumor cells to escape a hypoxic environment and pass through the blood circulation to new tissue environments. VEGF is currently known as the most potent growth factor in vascular endothelial cells. It can induce vascular endothelial cells proliferation and migration and enhance the permeability of blood vessels. It is an indispensable growth factor in angiogenesis. VEGF-A is a key member of VEGF family, which also includes VEGF-A, VEGF-B, VEGF-C, VEGF-D, VEGF-E, and placental growth factor (PGF). Studies have shown that VEGF is overexpressed in many tumors and downregulation of VEGF expression can inhibit tumor growth, invasion, and metastasis (Pavlidis and Pavlidis, 2013). VEGF can bind to the Vascular endothelial growth factor receptor (VEGF-R), causing abnormal changes in normal vascular endothelial cells, and promote tumor angiogenesis. VEGF-R can also activate tyrosine-protein kinase signaling to promote tumor cell proliferation and differentiation. VEGF receptors include VEGFR-1, VEGFR-2, and VEGFR-3, all of which have tyrosine kinase activity. Hirashima and others have found that VEGFR-1 and VEGFR-2 can be independent predictors of poor prognosis of tumor development (Hirashima et al., 2009). BEV is a VEGF-targeting monoclonal antibody and was the first drug to be approved for anti-tumor angiogenesis by the US Food and Drug Administration (FDA). It has become a first-line clinical treatment for patients with metastatic colorectal cancer. CD31 is highly expressed at the junction of vascular endothelial cells and is a marker molecule for tumor vascular endothelial cells and microvessels. The results of this study showed that CD31 protein was significantly lower in the subcutaneous xenograft tumors of the BEV, WCAF, and combination groups than in the control group (*p < 0.05*). The results suggest that WCAF can downregulate the expression of CD31 *in vivo* and produce synergistic anti-tumor activity in combination with BEV. In this study, we demonstrated that WCAF can inhibit the angiogenesis of human colon cancer HCT-116 xenografts and downregulate the expression of *VEGF-A* and VEGFR-1 protein. We also found that BEV treatment resulted in a decrease in the expression of *VEGF-A* in xenografts of human colon cancer HCT-116, but the expression of VEGFR-1 was unchanged. This observation may be because BEV targets the VEGF ligand instead of the tyrosine kinase VEGF receptor. In addition, Minjian et al. have found that BEV-treated colon cancer cells have increased expression of VEGF and bFGF *in vitro*. These cells also have reduced levels of ANG1, suggesting that BEV treatment may activate a feedback inhibition loop to regulate the expression of VEGF. This may account for the limited therapeutic efficacy of BEV in patients with advanced colon cancer (Zhao et al., 2017). Atsushi M et al. revealed that the expression of FGF2,FGFR2 was upregulated in the bevacizumab-treated tumor stroma. and this is related to acquired resistance to anti-angiogenic therapy with bevacizumab (Mitsuhashi et al., 2015). Our network pharmacology study indicated that FGF-2 was one of the top 10 genes that WCAF can regulate in clone cancer, the validation assay showed that the expression of FGF-2 were statistically significantly lower in the WCAF group and combination treatment group in comparison to the control group and BEV monotherapy group. Finally, we found that the expression of VEGFR-1 was statistically significantly lower in the combination treatment group in comparison to the BEV monotherapy group, suggesting that WCAF may be able to enhance the limited anti-angiogenic effects of BEV at the VEGF receptor level.

Obesity is an important risk factor for colorectal cancer (Bishehsari et al., 2014). Beccari et al. have found that Leptin signaling can serve as a new target for cancer therapy, especially for obese patients (Beccari et al., 2013). Studies have shown that Leptin can promote tumor cell proliferation, inhibit tumor cell apoptosis, and promote tumor angiogenesis (Ratke et al., 2010). Leptin is also closely related to VEGF and can upregulate VEGF expression and promote angiogenesis. In this study, we found that WCAF can downregulate the gene expression of Leptin and also Leptin-R protein in xenografts of HCT-116.

STATs are signal transducers and transcriptional activators, which are the focal points of a number of carcinogenic tyrosine kinases, such as growth factors, cytokines, and non-receptor tyrosine kinases. The over-activation of the signaling pathway can result in the aberrant expression of the downstream target genes, leading to the occurrence, development, and metastasis of colorectal cancer. STAT3 is an important member of the STATs family and can regulate a variety of key tumor factors, including the anti-apoptotic gene *BCL-2* and the cell cycle control gene *c-MYC*. These genes are downstream targets of the STAT3 signaling pathway and can promote cell proliferation, inhibit apoptosis, and promote tumor development and progression. STAT3 can also regulate angiogenesis in a variety of tumors and can also inhibit tumor invasion and metastasis. Baral et al. have found that activated STAT3 is associated with the occurrence of early colorectal cancer and can inhibit the apoptosis of colorectal cancer epithelial cells (Baral et al., 2009). Kusaba et al. have found that high expression of phosphorylated STAT3 (p-STAT3) can promote the invasion and metastasis of colorectal cancer, and the mechanism may be related to p-STAT3 regulation of tumor invasion, vascular invasion, and lymph node metastasis (Kusaba et al., 2005). Wei et al. have found that STAT3 can bind to the VEGF promoter, upregulate the expression of VEGF, and promote tumor metastasis (Wei et al., 2003). In this study, we found that WCAF can downregulate the expression of STAT3 and p-STAT3 proteins in xenografts of HCT-116 in nude mice, inhibit the STAT3 signaling pathway in colorectal cancer, and effectively inhibit tumor growth in combination with the anti-angiogenic agent BEV. BCL-2 is a factor that promotes tumor cell proliferation and inhibits tumor cell apoptosis and may be a downstream target gene for the STAT3 signaling pathway. The results of this study showed that there was no statistically significant difference in *BCL-2* expression in tumors from mice treated with vehicle, WCAF, BEV, and the combination of WCAF and BEV (*p* > 0.05). However, the expression of BCL-2 protein in each of the three treatment groups showed a decreasing trend in comparison to the control group. Our previous study has shown that Sijunzi Decoction, a component of Weichang’an can inhibit SGC-7901 cell proliferation by induce apoptosis by downregulating BCL-2 (Qian et al., 2016). In this study we observed that WCAF had a certain trend of inhibiting the expression of BCL-2 in colon cancer, which could be further studied in the future.

Antiangiogenic drugs combined with immune therapy are considered to have a synergistic effect on anti-tumor strategy. Simultaneous blockade of programmed death 1 and vascular endothelial growth factor receptor 2 (VEGFR2) induces synergistic anti-tumour effect *in vivo*. Our network pharmacology study indicated that WCAF can regulate immune related pathways include JAK-STAT signaling pathway, apoptosis, PI3K Akt signaling pathway and MAPK signaling pathway NF kappa B signaling pathway, T cell receptor signaling pathway and B cell receptor signaling pathway. Gato-Caas et al. (2015) and other studies have found that inhibition of AKT can limit *in vitro* MDSC differentiation, and Abu-Eid et al. (2014) discovered Treg is more susceptible to be inhibited after using AkT inhibitors, thus increasing CD8 + T cell number in tumor tissue to improve the control of tumor focus. In our study, we found WCAF can inhibited AKT1 expression in necrotic clone cancer tissue. leptin act as a negative regulator of NK cell functions and relate to increased cancer susceptibility in obesity *in vivo* experiment (Delort et al., 2015). leptin is involved in-obesity-induced carcinogenesis, tumor angiogenensis, cell proliferation and immune dysfunction. (Martí et al., 2001). In our study, WCAF treatment decreased the mRNA expression of Leptin and the protein level of LEP-R, in tumor tissues. Combined with BEV, WCAF showed a partial additive anti-tumor effect. The mechanism may also partitial related to immue regulation effection of WCAF targeting leptin signaling and AKT1.

## Conclusion

In summary, the JAK/STAT signaling pathway of Leptin can exert a critical role in tumor proliferation, apoptosis, and angiogenesis. WCAF inhibits HCT-116 tumor growth through inhibiting tumor angiogenesis and promoting tumor cell apoptosis. The mechanism may be related to Leptin/STAT3 signal transduction, VEGF-A, and VEGFR-1. In addition, WCAF makes up for the deficiency of BEV by inhibiting VEGFR-1and regulating tumor immunity. WCAF combined with BEV showed a partial additive anti-tumor effect in colon cancer *in vivo*. This may provide an experimental basis for the clinical application of the combination of TCM and anti-angiogenic therapy. There are also some limitations of our study. VEGFR2 is a very important receptor related to angiogenesis, the effect of WCAF on VEGFR2 needs further study. For a clear understanding of the underlying mechanism, we will perform an *in vitro* assay in the future. The critical ingredients also need to be studied and confirmed in a future study.

## Data Availability Statement

The datasets generated for this study are available on request to the corresponding author.

## Author Contributions

C-FP and XZ performed the experiments and drafted the manuscript. K-PS and LZ designed the experiments and revised the manuscript. J-WW and TY participated in the experiments and helped with data analysis. All authors read and approved the final manuscript.

## Funding

This work was supported by the National Natural Science Foundation of China (Grant no. 81503430) and The Sixth Batch of academic successor projects of Chinese traditional medicine experts in China (Grant no. (2017)29).

## Conflict of Interest

The authors declare that the research was conducted in the absence of any commercial or financial relationships that could be construed as a potential conflict of interest.

## References

[B1] Abu-Eid R. Samara R. N. Ozbun L. Abdalla M. Y. Berzofsky J. A. Friedman K. M. (2014). Selective inhibition of regulatory T cells by targeting the PI3K-Akt pathway. Cancer Immunol. Res. 2, 1080–1089. 10.1158/2326-6066.cir-14-0095 25080445 PMC4221428

[B2] Barabasi A. Albert R. (1999). Emergence of scaling in random networks. Science 286, 509–512. 10.1126/science.286.5439.509 10521342

[B3] Baral R. Bose A. Ray C. Paul S. Pal S. Haque E. (2009). Association of early phase of colorectal carcinogenesis with STAT3 activation and its relevance in apoptosis regulation. Exp. Mol. Pathol. 87, 36–41. 10.1016/j.yexmp.2009.03.002 19341726

[B4] Beccari S. Kovalszky I. Wade J. D. Otvos L. Jr. Surmacz E. (2013). Designer peptide antagonist of the leptin receptor with peripheral antineoplastic activity. Peptides 44, 127. 10.1016/j.peptides.2013.03.027 23567149

[B5] Benjamin L. E. Golijanin D. Itin A. Pode D. Keshet E. (1999). Selective ablation of immature blood vessels in established human tumors follows vascular endothelial growth factor withdrawal. J. Clin. Invest. 103. 159–165. 10.1172/JCI5028 9916127 PMC407882

[B6] Bindea G. Mlecnik B. Hackl H. Charoentong P. Tosolini M. Kirilovsky A. (2009). ClueGO: a Cytoscape plug-in to decipher functionally grouped gene ontology and pathway annotation networks. Bioinform. 25. 1091–1093. 10.1093/bioinformatics/btp101 PMC2666812 19237447

[B7] Bishehsari F. Mahdavinia M. Vacca M. Malekzadeh R. Mariani-Costantini R. (2014). Epidemiological transition of colorectal cancer in developing countries: environmental factors, molecular pathways, and opportunities for prevention. World J. Gastroenterol. 20, 6055–6072. 10.3748/wjg.v20.i20.6055 24876728 PMC4033445

[B8] Delort L. Rossary A. Farges M. Vasson M. Caldefie-Chézet F. (2015). Leptin, adipocytes and breast cancer: focus on inflammation and anti-tumor immunity. Life Sci. 140, 37–48. 10.1016/j.lfs.2015.04.012 25957709

[B9] Folkman J. (1971). Tumor angiogenesis: therapeutic implications. N. Engl. J. Med. 285, 1182–1186. 10.1056/NEJM197111182852108 4938153

[B10] Gato-Caas M. de Morentin X. M. Blanco-Luquin I. Fernandez-Irigoyen J. Zudaire I. Liechtenstein T. (2015). A core of kinase-regulated interactomes defines the neoplastic MDSC lineage. Oncotarget. 6, 27160–27175. 10.18632/oncotarget.4746 26320174 PMC4694980

[B11] Gerger A. Labonte M. Lenz H. J. (2011). Molecular predictors of response to antiangiogenesis therapies. Canc. J. 17, 134. 10.1097/PPO.0b013e318212db3c 21427557

[B12] Gu Y. Han Y. Y. Zheng J. Yang J. K. (2006). Analysis of therapeutic effect of Wei Chang An in colorectal cancer. liaoning zhongyiyao daxue xuebao 8, 5. 10.3969/j.issn.1673-842X.2006.05.001

[B13] He M. Hou J. Wang L. Zheng M. Fang T. Wang X. (2017). Actinidia chinensisPlanch root extract inhibits cholesterol metabolism in hepatocellular carcinoma through upregulation ofPCSK9. Oncotarget. 8, 42136–42148. 10.18632/oncotarget.15010 28178673 PMC5522055

[B14] Hirashima Y. Yamada Y. Matsubara J. Takahari D. Okita N. Takashima A. (2009). Impact of vascular endothelial growth factor receptor 1, 2, and 3 expression on the outcome of patients with gastric cancer. Canc. Sci. 100, 310. 10.1111/j.1349-7006.2008.01020.x PMC11159319 19068081

[B15] Hua L. Shoudan L.. (2009). Local network topology in human protein interaction data predicts functional association. PloS One 4, e6410. 10.1371/journal.pone.0006410 19641626 PMC2713831

[B16] Kim K. J. Li B. Winer J. Armanini M. Gillett N. Phillips H. S. (1993). Inhibition of vascular endothelial growth factor-induced angiogenesis suppresses tumour growth *in vivo* . Nature 362, 841. 10.1038/362841a0 7683111

[B17] Kong L. H. Shen K. P. Pan C. F. (2019). Effects of Weichangan Recipe on leptin-stimulated proliferation and secretion of VEGF in colon cancer HT-29 cells. Chin J. Integr. Trad. West Med. Dig. 27, 199–202. 10.3969/j.issn.1671-038X.2019.03.08

[B18] Kusaba T. Nakayama T. Yamazumi K. Yakata Y. Yoshizaki A. Nagayasu T. (2005). Expression of p-STAT3 in human colorectal adenocarcinoma and adenoma; correlation with clinicopathological factors. J. Clin. Pathol. 58, 833. 10.1136/jcp.2004.023416 16049285 PMC1770863

[B19] Lu Y. L. Shen K. P. (2015). Clinical experience of professor Qiu Jiaxin in treating gastrointestinal carcinoma by invigorating spleen method. Acta Univ. Tradit. Med Sin. Pharmacol. Shanghai 29. 1–3. 10.16306/j.1008-861x.2015.01.001

[B20] Malonne H. Langer I. Kiss R. Atassi G. (1999). Mechanisms of tumor angiogenesis and therapeutic implications: angiogenesis inhibitors. Clin. Exp. Metastasis 17. 1–18. 10390141 10.1023/a:1026443925807

[B21] Martí A. Marcos A. Martínez J. A. (2001). Obesity and immune function relationships. Obes Rev. 2, 131–140. 10.1046/j.1467-789x.2001.00025.x 12119664

[B22] Mitsuhashi A. Goto H. Saijo A. Trung V. T. Nishioka Y. (2015). Fibrocyte-like cells mediate acquired resistance to anti-angiogenic therapy with bevacizumab. Nat. Commun. 6. 8792. 10.1038/ncomms9792 26635184 PMC4686833

[B23] Pan C. F. Liang W. M. Gu X. Hu B. Shen K. p. (2009). Effects of“Weichang’an decotion” on growth, Metastasis and angiogenesis of gastric cancer and MMP2 expression. Acta univ.Tradit. med. sinensis pharmacol. shanghai 23, 64–66. 10.16306/j.1008-861x.2009.01.018

[B24] Pavlidis E. T. Pavlidis T. E. (2013). Role of bevacizumab in colorectal cancer growth and its adverse effects: A review. World J. Gastroenterol. 19, 5051–5060. 10.3748/wjg.v19.i31.5051 23964138 PMC3746376

[B25] Qian J. Li J. Jia J. Jin X. Yu D. Guo C. (2016). Different concentrations of sijunzi decoction inhibit proliferation and induce apoptosis of human gastric cancer SGC-7901 side population. Afr. J. Tradit., Complementary Altern. Med. 13. 145–156. 10.21010/ajtcam.v13i4.19 PMC5566137 28852730

[B26] Ratke J. Entschladen F. Niggemann B. Zänker K. S. Lang K. (2010). Leptin stimulates the migration of colon carcinoma cells by multiple signaling pathways. Endocr. Relat. Canc. 17. 179–189. 10.1677/ERC-09-0225 19952122

[B27] Saltz L. B. Clarke S. Díazrubio E. Scheithauer W. Figer A. Wong R. (2008). Bevacizumab in combination with oxaliplatin-based chemotherapy as first-line therapy in metastatic colorectal cancer: a randomized phase III study. J. Clin. Oncol. 26, 2013–2019. 10.1200/JCO.2007.14.9930 18421054

[B28] Shen K. P. Pan C. F. (2007). Clinical study on the effect of Weichang'an recipe in preventiong metastasis of gastriccancer and invitro study of its effect on invasion and migration capacities of MGC-803 cells. J.Modern Oncology 15, 1301–1303. 10.3969/j.issn.1672-4992.2007.09.037

[B29] Shen K. P. Wang H. Y. Bing H. U. Liu W. Shuang-Yang H. U. (2012). Antitumor effect of weichangan decoction on subcutaneously implanted human gastric cancer in nude mice. Liaoning J. Trad. Chinese Med. 39, 215–217. 10.13192/j.ljtcm.2012.02.28.shenkp.011

[B30] Tao L. Yang J. K. Gu Y. Zhou X. Zhao A. G. Zheng J. (2015). Weichang’an and 5-fluorouracil suppresses colorectal cancer in a mouse model. World J. Gastroenterol. 21. 1125–1139. 10.3748/wjg.v21.i4.1125 25632185 PMC4306156

[B31] Tao L. Ying G. U. Bin W. U. Chen B. Zhao A. G. (2013). Effect of "Weichang'an Decoction" on growth and hepatic metastasis of orthotopic transplant in nude mouse. Shanghai J. Tradit. Chin. Med. 47, 79–84. 10.16305/j.1007-1334.2013.04.029

[B32] Voest E. E. D'Amore P. A. (2001). Tumor angiogenesis and microcirculation. Int. J. Hematol. 74, 479. 10.1007/BF02982099 29349752

[B33] Wei D. Le X. L. Wang L. Frey J. A. Gao A. C. Peng Z. (2003). Stat3 activation regulates the expression of vascular endothelial growth factor and human pancreatic cancer angiogenesis and metastasis. Oncogene 22, 319–329. 10.1038/sj.onc.1206122 12545153

[B34] Wolpin B. M. Mayer R. J. (2008). Systemic treatment of colorectal cancer. Gastroenterology 134. 1296–1310. 10.1053/j.gastro.2008.02.098 18471507 PMC2528832

[B35] Zhao A. G. Yang J. K. You S. F. Li T. Zhao H. L. Gu Y. (2007). [Difference of gene expression profile in human gastric cancer grafted onto nude mice treated with WCA]. Zhongguo Zhong Yao Za Zhi 32. 2028–2036. 10.3321/j.issn:1001-5302.2007.19.018 18161298

[B36] Zhao M. Yu Z. Li Z. Tang J. Lai X. Liu L. (2017). Expression of angiogenic growth factors VEGF, bFGF and ANG1 in colon cancer after bevacizumab treatment *in vitro*: a potential self-regulating mechanism. Oncol. Rep. 37, 601. 10.3892/or.2016.5231 27840995

[B37] Zhou H. Shen K. P. (2009). Clinical study on ”WeiChangAn” in treating postoperative colorectal carcinomaof spleen deficiency. SH.J.TCM 43, 36–39. 10.16305/j.1007-1334.2009.06.033

