# Supplementary figures and images for: Weichang’an Formula Inhibits Tumor Growth in Combination with Bevacizumab in a Murine Model of Colon Cancer—Making up for the Deficiency of Bevacizumab by inhibiting VEGFR-1
Source: Front Pharmacol. 2020 Nov 30;11:512598. doi: 10.3389/fphar.2020.512598 (PMC7970919; doi:10.3389/fphar.2020.512598)

**Leptin/stat3 signaling pathway**


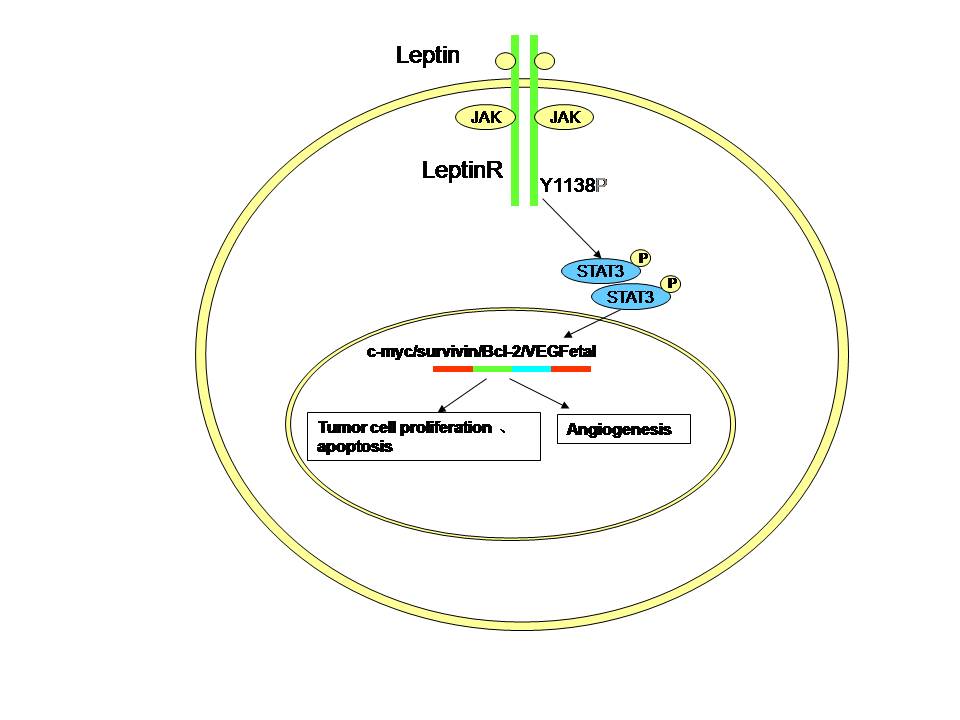


**Brief graph of this research**


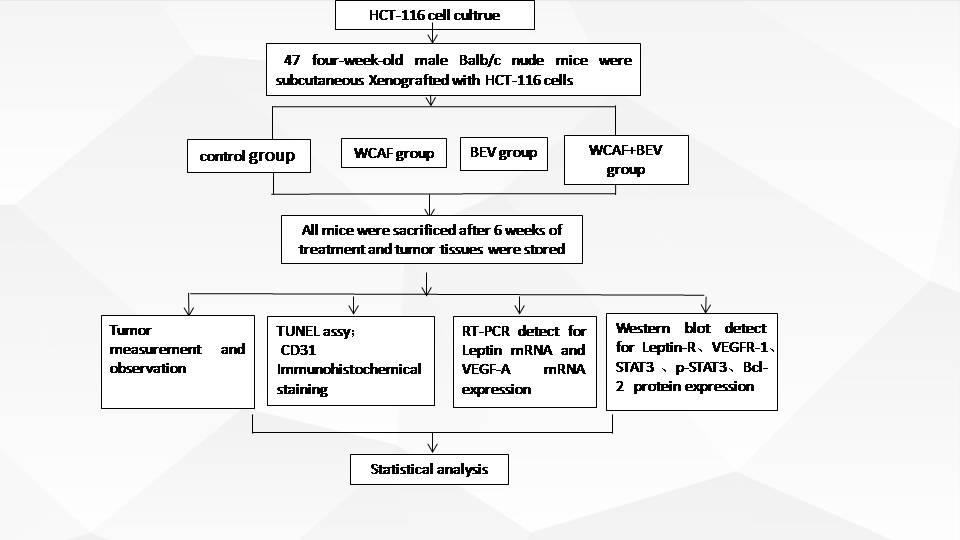

Supplement: Supplementary file 1 [file datasheet1.docx]
